# Aggression and social experience: genetic analysis of visual circuit activity in the control of aggressiveness in *Drosophila*

**DOI:** 10.1186/s13041-014-0055-0

**Published:** 2014-08-13

**Authors:** Mahmoudreza Ramin, Claudiu Domocos, David Slawaska-Eng, Yong Rao

**Affiliations:** 1 McGill Centre for Research in Neuroscience, McGill University Health Centre, 1650 Cedar Avenue, Montreal H3G 1A4, Quebec, Canada; 2 Department of Neurology and Neurosurgery, Integrated Program in Neuroscience, McGill University Health Centre, 1650 Cedar Avenue, Montreal H3G 1A4, Quebec, Canada; 3 Department of Medicine, McGill University Health Centre, 1650 Cedar Avenue, Montreal H3G 1A4, Quebec, Canada

## Abstract

**Background:**

Animal aggressiveness is controlled by genetic and environmental factors. Among environmental factors, social experience plays an important role in modulating aggression in vertebrates and invertebrates. In *Drosophila*, pheromonal activation of olfactory neurons contributes to social suppression of aggression. While it was reported that impairment in vision decreases the level of aggression in *Drosophila*, it remains unknown if visual perception also contributes to the modulation of aggression by social experience.

**Results:**

In this study, we investigate the role of visual perception in the control of aggression in *Drosophila*. We took several genetic approaches to examine the effects of blocking visual circuit activity on fly aggressive behaviors. In wild type, group housing greatly suppresses aggressiveness. Loss of vision by mutating the *ninaB* gene does not affect social suppression of fly aggression. Similar suppression of aggressiveness by group housing is observed in fly mutants carrying a mutation in the *eya* gene leading to complete loss of eye. Chronic visual loss does not affect the level of aggressiveness of single-housed flies that lack social experience prior to behavioral tests. When visual circuit activity is acutely blocked during behavioral test, however, single-housed flies display higher levels of aggressiveness than that of control flies.

**Conclusion:**

Visual perception does not play a major role in social suppression of aggression in *Drosophila*. For single-housed individuals lacking social experience prior to behavioral tests, visual perception decreases the level of aggressiveness.

## Background

Aggression is an innate behavior that allows animals to compete for limited resources, such as food, mating partners and habitats. The level of aggressiveness is influenced by both genetic and environmental factors [1]. Accumulated evidence supports that social experience is one of the most important environmental factors that affect aggression in humans [2], rats [3]–[5] and *Drosophila*[6].

Recent studies have shed light on molecular mechanisms underlying the control of aggression by social experience. For instance, Cyp6a20, a cytochrome P450, is identified as a common genetic target for the control of aggressiveness by social experience in *Drosophila*[7]. It has also been reported that chronic activation of Or65a olfactory neurons by the volatile pheromone 11-cis-vaccenyl acetate (cVA) contributes to social suppression of aggressiveness in *Drosophila*[8],[9]. However, it remains unknown if other sensory stimuli such as vision, also contributes to social suppression of aggression in *Drosophila*.

A previous study reports that mutations in the *white (w)* gene that regulates eye pigmentation, greatly decrease aggressiveness of single-housed flies, suggesting that vision is required for normal aggression [10]. To determine if visual perception contributes to social suppression of aggressiveness, we investigated if the blockade of visual circuit activity affects social suppression of aggression. We also examined the effects of visual impairment on aggressiveness of single-housed flies that lack social experience prior to behavioral tests.

## Results

### Loss of vision in *ninaB* mutants does not prevent social suppression of aggression

**Figure 1:**
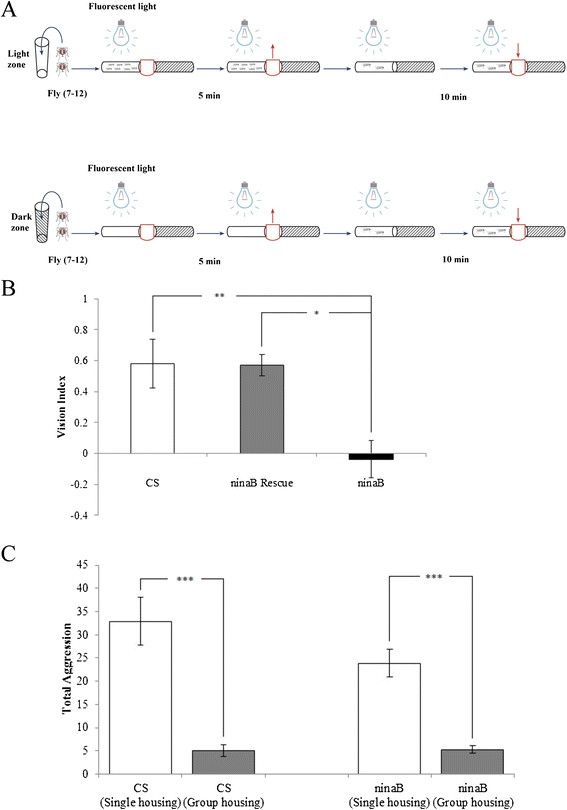
**Visual impairment in*ninaB*mutants does not affect social suppression of aggressiveness. (A)** Schematic drawing of phototaxis assay (see Materials and methods). **(B)** Vision index of flies was quantified (see Materials and methods). Canton-S (CS) wild-type flies prefer to stay in light zone. Whereas *ninaB*^1^ mutant flies distributed randomly in light and dark zones, indicating impairment in vision. Rescued flies in which a *UAS-ninaB* transgene was expressed in photoreceptors in *ninaB*^1^ mutant flies under control of the eye-specific *GMR-GAL4* driver, showed light preference similar to that of wild-type flies. ^**^p < 0.01, ^*^p < 0.05. Number of experiments performed: CS, n = 11; *ninaB*^1^ Rescue, n = 10; *ninaB*^1^, n = 11. **(C)** Social suppression of aggressiveness of wild-type and *ninaB*^1^ mutant flies. The level of aggressiveness (i.e. total aggression) was quantified by counting the number of all aggressive events (i.e. lunges, wing threats, tussles, boxing, and holding) within 10-min period. Pairs of flies tested: CS, n = 27 (single housing), n = 21 (group housing); *ninaB*^1^ mutants, n = 22 (single housing), n = 20 (group housing). ^***^p < 0.0001. Error bars represent SEM.

To determine if visual perception contributes to social suppression of fly aggression, we examined if the modulation of aggressiveness by social experience is affected in blind *ninaB* mutant flies. *ninaB* encodes a β,β-carotene-15,15′-dioxygenase that mediates the generation of visual chromophores [11]. To confirm that *ninaB*^1^ mutation causes loss of vision [12], we performed phototaxis experiments similarly as described previously [13]. For each experiment, ~7-12 flies were aspirated into dark or light zone, and then allowed to move freely (Figure 1A). Wild-type flies or rescue flies in which a *ninaB* transgene was expressed in *ninaB*^1^ mutants show a preference for light zone (Figure 1B). By contrast, *ninaB*^1^ mutants selected light or dark zone randomly. This result confirms that vision is impaired in *ninaB*^1^ mutants.

We then performed experiments to examine the level of aggressiveness in flies with or without social experience. Wild-type flies reared in isolation (single housing) displays a much higher level of aggressiveness compared to flies reared in group (group housing) (Figure 1C), indicating that social experience prior to aggression assays suppresses the level of aggressiveness. Similar to that of wild-type flies, group housing greatly decreased the level of aggressiveness of *ninaB*^1^ mutants (Figure 1C). This result suggests that visual perception does not contribute significantly to social suppression of fly aggressiveness.

### Complete loss of eye does not prevent social suppression of aggression

**Figure 2:**
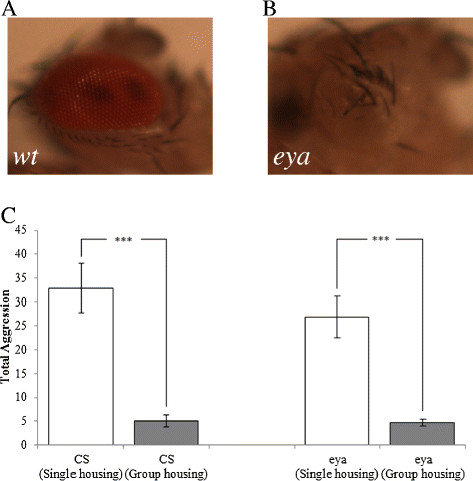
**Complete loss of eye does not affect social suppression of aggressiveness. (A)** The compound eye consists of ~800 ommatidia in wild type. **(B)** In *eya*^2^ mutants, the eye is completely absent. **(C)** Complete loss of eye in *eya*^2^ mutants did not affect social suppression of aggressiveness. ^***^p < 0.0001. Pairs of flies tested: CS, n = 27 (single housing), n = 21 (group housing); *eya*^2^ mutants, n = 20 (single housing), n = 20 (group housing). Error bars represent SEM.

To further confirm above result, we also examined if complete loss of eye in the *eyes absent* gene (*eya*) mutants affects social suppression of aggression. Mutations in the *eya* gene cause defects in eye development [14], leading to loss of eye (Figure 2B). Like that of wild-type flies, we found that the level of aggressiveness of *eya* mutants was greatly suppressed by social experience prior to aggression assays (Figure 2C). This result, together with the result from testing *ninaB*^1^ mutants (Figure 1C), argue against a major role for visual perception in mediating social suppression of fly aggressiveness.

### Chronic visual loss does not affect aggressiveness of single-housed flies lacking social experience prior to behavioral assays

When we examined the effects of chronic visual loss on social suppression of aggression, we found that single-housed flies in which vision is impaired still showed high levels of aggressiveness (Figures 1 and 2). Such results are in marked contrast to a previous report that suggests that visual impairment greatly decreases aggressiveness of single-housed flies, based on analysis of white-eyed flies carrying mutations in the *w* gene [10]. To further test the potential role of visual perception in regulating aggressiveness of single-housed flies, we performed more detailed analysis of flies with chronic visual loss.

**Figure 3:**
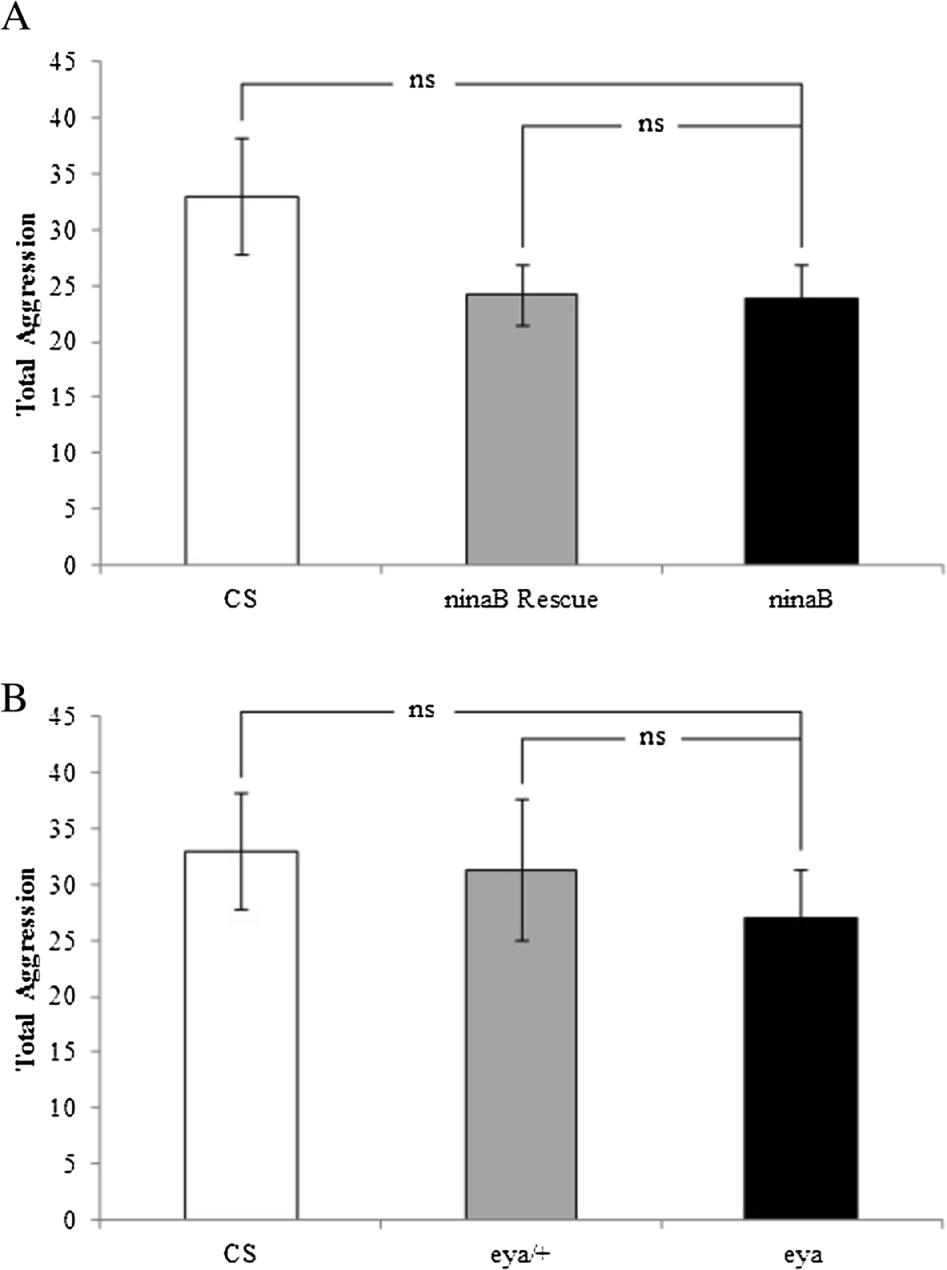
**Chronic visual loss does not affect aggressiveness of single-housed flies that lack social experience prior to behavioral tests. (A)** The level of aggressiveness (i.e. total aggression) was quantified by counting the number of all aggressive events (i.e. lunges, wing threats, tussles, boxing, and holding) within 10-min period. No significant difference was observed between single-housed wild type and *ninaB*^1^ mutants, or between single-housed *ninaB*^1^ mutants and rescued individuals in which vision was restored by eye-specific expression of a *ninaB* transgene in *ninaB*^1^ mutants. Pairs of flies tested: CS, n = 27; *ninaB*^1^ Rescue, n = 26; *ninaB*^1^, n = 22. **(B)** Loss of eye in *eya*^2^ mutants did not affect aggressiveness of single-housed flies. The level of aggressiveness of single-housed *eya*^2^ mutants was comparable to that of wild-type or *eya*^2^ heterozygous flies. Pairs of flies tested: CS, n = 27; *eya*^2^/+, n = 20; *eya*^2^, n = 20. ns, not significant (p > 0.05). Error bars represent SEM.

The level of aggressiveness of isolated *ninaB* mutant flies was compared to that of wild-type or rescue flies in which vision was restored in *ninaB* mutants by eye-specific expression of a *ninaB* transgene. No significant difference in the levels of aggressiveness was observed between blind flies (i.e. *ninaB* mutants) and flies with normal vision (i.e. wild-type or rescue flies) (Figure 3A). Similar results were observed when the level of aggressiveness of single-housed *eya* flies in which the eye is absent was compared to that of wild-type or *eya* heterozygous flies with intact eye (Figure 3B). These results confirm that chronic visual loss does not affect the levels of aggressiveness of single-housed flies.

**Figure 4:**
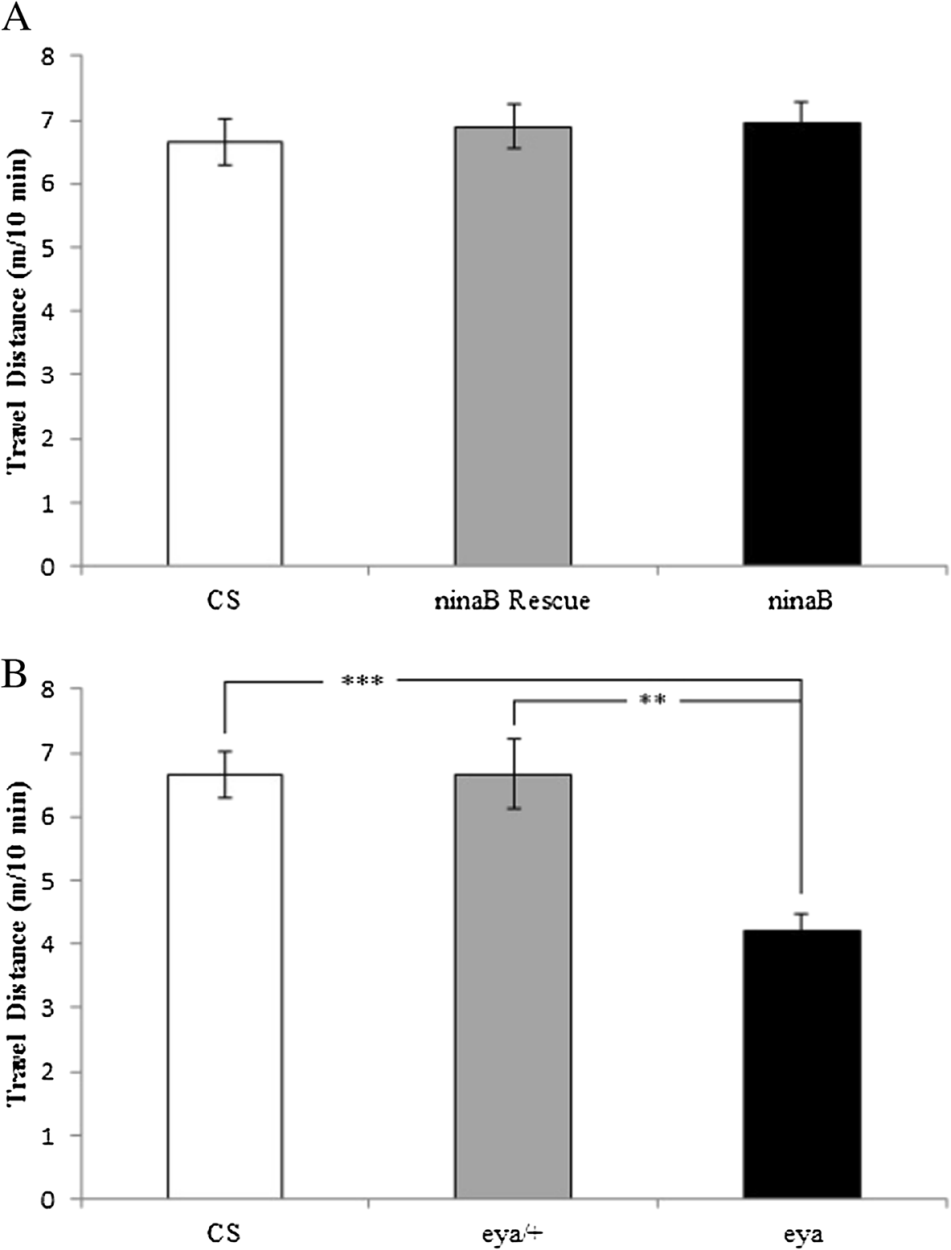
**The effects of chronic visual impairment on fly locomotor activity. (A)** Travel distance of flies within 10-min period was measured. No significant difference in locomotion was observed between *ninaB*^1^ and wild-type flies or between *ninaB*^1^ and rescued flies. Pairs of flies tested: CS, n = 30; *ninaB*^1^ Rescue, n = 22; *ninaB*^1^, n = 30. **(B)** Travel distance of *eya*^2^ homozygous mutant flies was measured. Compared to that of wild-type or *eya*^2^ heterozygous flies, lower locomotor activity was observed in *eya*^2^ homozygous mutant flies. Pairs of flies tested: CS, n = 30; *eya*^2^/+, n = 29; *eya*^2^, n = 26. ^**^p < 0.01, ^***^p < 0.001. Error bars represent SEM.

To determine if chronic visual loss affects locomotor activity, we examined travel distance of wild-type, *ninaB* mutant or rescued flies within 10-minute period. No significant difference in travel distance was observed (Figure 4A). We also examined travel distance of *eya* heterozygous and homozygous flies. While loss of vision in *eya* homozygous mutants does not affect aggressiveness of isolated flies (Figure 3B), the locomotor activity of *eya* mutants was lower than that of wild-type or *eya* heterozygous mutants (Figure 4B).

### Acute blockade of visual circuit activity increases aggressiveness of single-housed flies

We then examined if temporal blockade of visual circuit activity during the period of aggression assays affects aggressiveness of flies that were single-housed prior to behavioral assay. To test this, synaptic transmission from photoreceptor cells was temporally blocked by eye-specific expression of a temperature-sensitive form of *shibire* (*shi*^ts^) that encodes the fly homolog of dynamin. This allows the blockade of synaptic transmission in photoreceptor cells at restrictive temperature [15],[16].

**Figure 5:**
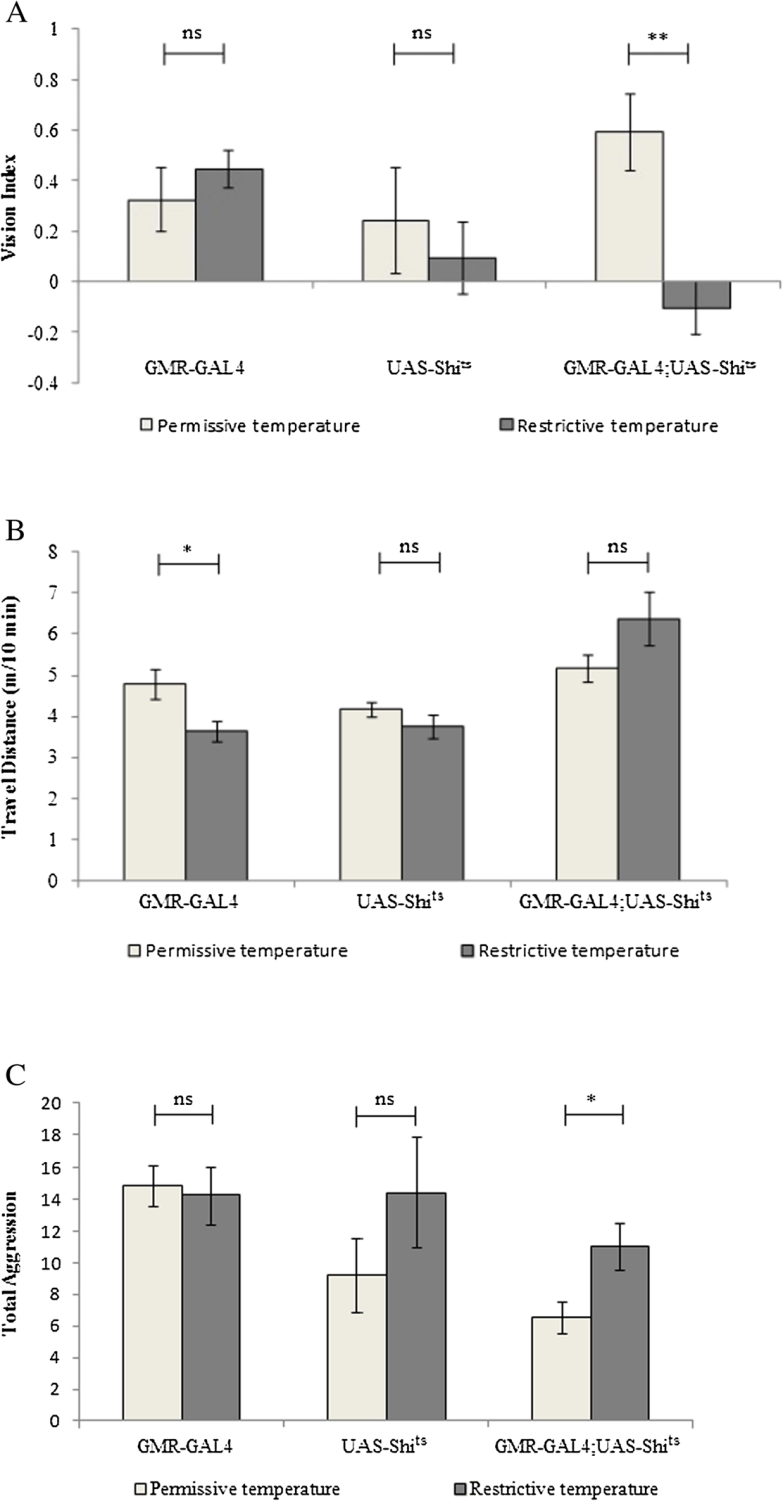
**Temporal blockade of visual circuit activity increases aggressiveness of single-housed flies.***UAS*-*Shi*^ts^ were expressed under control of eye-specific driver *GMR-GAL4*, which blocks synaptic transmission in photoreceptor cells at restrictive temperature (32°C). Flies that only carry *GMR-GAL4* or *UAS*-*Shi*^ts^ were used as controls. Vision index **(A)**, locomotor activity **(B)** and aggression **(C)** of flies were examined. The performance at restrictive temperature (32°C) was compared to that of same-genotype flies at permissive temperature (22°C). **(A)** Blockade of photoreceptor synaptic transmission impaired fly vision. ^**^p < 0.01. “ns” indicates p > 0.05. Number of experiments performed at permissive temperature: *GMR-GAL4*, n = 9; *UAS*-*Shi*^ts^, n = 10; *GMR-GAL4*;*UAS*-*Shi*^ts^, n = 9. Number of experiments performed at restrictive temperature: *GMR-GAL4*, n = 10; *UAS*-*Shi*^ts^, n = 8; *GMR-GAL4*;*UAS*-*Shi*^ts^, n = 9. **(B)** Travel distance of flies within 10-min period was measured. ^*^p < 0.05. Pairs of flies tested at permissive temperature: *GMR-GAL4*, n = 20; *UAS*-*Shi*^ts^, n = 20; *GMR-GAL4*;*UAS*-*Shi*^ts^, n = 20. Pairs of flies tested at restrictive temperature: *GMR-GAL4*, n = 20; *UAS*-*Shi*^ts^, n = 21; *GMR-GAL4*;*UAS*-*Shi*^ts^, n = 19. **(C)** Aggressiveness of single-housed flies in which photoreceptor synaptic transmission was temporally blocked at restrictive temperature was compared to that of flies at permissive temperature. ^*^p < 0.05. Pairs of flies tested at permissive temperature: *GMR-GAL4*, n = 20; *UAS*-*Shi*^ts^, n = 20; *GMR-GAL4*;*UAS*-*Shi*^ts^, n = 20. Pairs of flies tested at restrictive temperature: *GMR-GAL4*, n = 20; *UAS*-*Shi*^ts^, n = 21; *GMR-GAL4*;*UAS*-*Shi*^ts^, n = 19. Error bars represent SEM.

A shift from permissive temperature (i.e. 22°C) to restrictive temperature (i.e. 32°C ) effectively blocked visual circuit activity, leading to loss of vision at restrictive temperature (Figure 5A). Blockade of visual circuit activity, however, did not affect locomotor activity (Figure 5B). We then examined the effects of temporally blocking visual circuit activity on the level of aggressiveness. Compared to that of flies at permissive temperature, the level of aggressiveness of single-housed flies at restrictive temperature increased significantly (Figure 5C). This result suggests that visual perception helps decrease aggressiveness of single-housed flies.

## Discussion

Social suppression of aggression is observed in both vertebrates and invertebrates. While pheromonal activation of olfactory neurons has been implicated in this process in *Drosophila*, it remains unclear if other sensory cues also contribute to the modulation of aggressiveness by social experience. In this study, we investigated the effects of manipulating visual circuit activity in the control of fly aggression. We showed that blockade of visual circuit activity does not prevent social suppression of aggression. While chronic blockade of visual circuit activity does not affect aggressiveness of single-housed flies that lack social experience prior to behavioral tests, acute blockade of visual circuit activity increases the level of aggressiveness of single-housed flies.

Our results indicate that visual perception is not a major factor that allows male flies to recognize and interact with each other for suppressing aggressiveness by social experience. Whereas pheromonal activation of certain neurons in the olfactory system has been shown to contribute significantly to social suppression of aggression [8],[9]. Recent studies showed that the gustatory system also plays a role in modulating fly aggression [17]–[19]. Future studies are required to determine if gustatory cues contribute to social suppression of fly aggressiveness.

Our result showing that acute blockade of visual circuit activity increases the level of aggressiveness of single-housed flies is surprising. Previous work by Heisenberg and colleagues showed that mutants defective in the *w* gene display much lower levels of aggressiveness [10]. Since the *w* gene mediates eye pigmentation, this result suggests that visual perception promotes aggressiveness. However, since removing *w* gene in the brain also causes a decrease in aggression [10], together with that white-eyed cricket mutants display normal levels of aggressiveness [20], we speculate that the decrease in the level of aggressiveness of white-eyed flies may not be caused by vision impairment.

While acute blockade of visual circuit activity increases the level of aggressiveness of isolated flies, chronic blockade of visual circuit activity does not affect fly aggressiveness. One possible explanation is that chronic blockade of visual circuit activity increases the sensitivity of fly response to other sensory cues, which may compensate for loss of visual perception in decreasing aggressiveness. Future studies are required to address these possibilities.

## Conclusion

Visual circuit activity does not contribute significantly to social suppression of aggression in *Drosophila*. For individuals reared in isolation and thus lack social experience prior to behavioral tests, however, visual perception helps decrease the level of aggressiveness.

## Materials and methods

### Stocks and rearing condition

*ninaB*^1^ and *eya*^2^ mutants were obtained from Bloomington stock center. *ninaB* rescue experiments were performed by generating *GMR-GAL4*/+; *ninaB*^1^, *UAS*-*ninaB*/*ninaB*^1^, *UAS*-*ninaB* flies. *UAS*-*Shi*^ts^ were provided by Dr. Greg Suh (NYU). *GMR-GAL4*;*UAS-Shi*^ts^ flies were generated by crossing male *UAS-Shi*^ts^ with female *GMR-GAL4* flies. *Canton-S* flies were used as wild-type controls. Flies were reared at 25°C with 50-60% humidity and 12 hour light–dark cycle. Newly emerged males from pupal cases were single-housed in a 2 ml microfuge tube containing 1 ml of fly food for 6 days prior to aggression and locomotion assays. For experiments testing social influence of fly aggressiveness, flies were grouped in vials (10 flies per vial) and reared for 6 days prior to aggression assay. For vision tests, flies were group housed (7–12 flies) per vial and reared for 5–7 days prior to vision tests.

### Vision assay

To examine fly vision, we used standard vials with 1.2 cm radius and 9.5 cm height. One vial was completely covered with dark duct tape except for the tip where flies were aspirated, and was indicated as dark zone. Another uncovered transparent vial was indicated as light zone. The two vials were attached together, separated by a paper cardboard, and horizontally placed under a light source (Figure 1A). For each experiment, ~7-12 flies were gently aspirated into either dark or light zone. Flies were allowed to get accustomed to new environment for 5 min. The cardboard was then removed gently in a way that did not agitate the flies. Flies were let freely move between light and dark zone for 10 min. The number of flies in light zone and dark zone was counted. Vision index is defined as: (number of light-zone flies – number of dark-zone flies)/total number of flies.

### Aggression assay

Aggression assay was performed by placing a pair of male flies in a circular fighting chamber (7 mm radius and 3.5 mm height), which has a central pad (4 mm radius) covered with food*,* and outer space filled with agar to reduce the dehydration of food. Behavioral tests were carried out at 22°C. For experiments involving acute blockade of visual circuit activity, aggression tests were performed at 22°C (permissive temperature) or 32°C (restrictive temperature). Two male flies of the same genotype were gently aspirated to the fighting chamber. After 5 minutes for flies to get accustomed to the environment, their behaviors were recorded with a high definition (HD) camera under fluorescent lamp for 10 minutes. The total number of aggressive events (i.e. lunges, wing threats, tussles, boxing, and holding) per 10-min period was used to indicate the level of aggressiveness.

### Locomotion assay

Movement of two flies in a small round chamber was videotaped and analyzed by CADABRA software [21]. Two flies were gently aspirated into a chamber similar to the fighting chamber used for aggression assay. Movement of flies was recorded for 10 min.

### Statistical analysis

Data were expressed as mean ± SEM and processed by commercially available GraphPad Prism® 5.0. Mann Whitney test, or Kruskal-Walis ANOVA test followed by Dunn’s multiple comparison test were used in statistical analysis. p-value less than 0.05 (p < 0.05) is considered as significant.

## Competing interests

The authors declare that they have no competing interests.

## Authors’ contributions

MR conducted the experiments, and was involved in writing the manuscript. CD and DS helped data quantitation. YR supervised the project and wrote the manuscript. All authors read and approve the manuscript.
